# Human Papillomavirus Type 16 *E1* Mutations Associated with Cervical Cancer in a Han Chinese Population

**DOI:** 10.7150/ijms.34279

**Published:** 2019-06-24

**Authors:** Yueting Yao, Zhiling Yan, Shuying Dai, Chuanyin Li, Longyu Yang, Shuyuan Liu, Xinwen Zhang, Li Shi, Yufeng Yao

**Affiliations:** 1 Institute of Medical Biology, Chinese Academy of Medical Sciences & Peking Union Medical College, Kunming 650118, Yunnan, China; 2 Department of Gynaecologic Oncology, The 3rd Affiliated Hospital of Kunming Medical University, Kunming 650118, China.; 3 School of Basic Medical Science, Kunming Medical University, Kunming 650500, China.

**Keywords:** Human papillomavirus type 16 (HPV16), *E1* gene, Cervical cancer, Mutations, Association study

## Abstract

Human papillomavirus type 16 (HPV16) is a high-risk HPV type and a potent carcinogen. HPV E1 is one of the most highly conserved proteins and it plays a central role in initiating HPV DNA replication. In current study, we enrolled 161 HPV16-positive cervical cancer patients (case group) and 171 HPV16-positive asymptomatic individuals (control group) in a study to analyse the association between HPV16 *E1* genetic mutations and cervical cancer. The samples of case group were cervical cancer tissues and the samples of control group were cervical exfoliated cells. Three variants (A4, A1-A3 and D3) were found in the case group, 68.3% of the HPV16 *E1* sequences belonged to the A4 (As) sub-lineage, 29.2% belonged to the A1-A3 (EUR) sub-lineage, and 2.5% belonged to the D3 (AA1) sub-lineage. Two variants (A4 and A1-A3) occurred in the control group. The A4 (As) sub-lineage was predominant in this group as well (66.1%), followed by the A1-A3 (EUR) sub-lineage (33.9%), but the D3 (AA1) sub-lineage was not found in the control group. The distribution of the HPV16 variants between the case and control groups was significantly different (*P*<0.05). When the distribution of the HPV16 *E1* gene mutations was compared, the distribution of twenty-seven mutations was significantly different between the case and control groups (*P*<0.05), and twenty-two mutations occurred only in the D3 (AA1) sub-lineage, two were found only in the A4 (As) sub-lineage, one was found in the A1-A3 (EUR) sub-lineage, two was found in both the A4 (As) and A1-A3 (EUR) sub-lineages. In the sub-lineage analysis, the differences in the T933A (A23A), T1014G (D50E) and G2160A (R432R) mutations were statistically significant between the case and control groups for the A4 (As) sub-lineage (*P*<0.05), and the differences in the T2232C (F456F), G2337A (M491I) and A2547G (P561P) mutations were statistically significant between the case and control groups for the A1-A3 (EUR) sub-lineage (*P*<0.05). In the current study, we describe specific mutations in the HPV16 *E1* gene associated with cervical cancer, and our study will provide a good reference for further functional studies of the relationship between cervical cancer carcinogenesis and HPV genes.

## Introduction

Cervical cancer is one of the most common malignancies in females. The major factor contributing to the development of cervical cancer is persistent infection with high-risk human papillomaviruses (HPVs) 1, 2. HPVs could be divided into low risk and high-risk types. Low risk HPVs are associated with 90% genital warts and also related to the development of recurrent respiratory papillomatosis, including HPV6 and HPV11 3-5. High-risk HPVs are frequently associated with cervical cancer and approximately 15 HPV genotypes (HPV16, 18, 31, 33, 35, 39, 45, 51, 52, 56, 58, 59, 68, 73, and 82) are recognized as high-risk types according to an epidemiological survey 6. Among these high-risk types, HPV16 is the most common type detected in patients with cervical cancer worldwide and a prospective cohort study showed that it was also the most persistent HPV type during infection with HPVs 7, 8. In addition, HPV16 also thought to be associated with head and neck squamous cells carcinomas 9, 10.

The HPV16 genome is a circular, double-stranded 7,908 bp molecule and is divided into three major regions: the early region, encoding viral early proteins, compring the E1, E2, E4, E5, E6 and E7 genes; the late region, encoding the viral late proteins L1 and L2; and the long control region (LCR), which includes the viral transcription control sequence and the replication sequence 11. HPV E1 and E2 are necessary for viral replication. The E1 protein recognizes and binds to an AT-rich sequence within the viral DNA origin of replication in cooperation with the E2 protein and the complex composed of the E1 and E2 proteins then binds to the viral origin of replication with high affinity and initiates DNA replication via the E1 ATP-dependent helicase domain 12. Furthermore, the E1 helicase domain interacts with DNA polymerase a-primase, topoisomerase I and the single-stranded DNA-binding protein RPA to recruit cellular replication factors to the viral origin of replication, thus forming an active replication complex 13-16. In 2017, Baedyananda *et al*. reported that the high expression of HPV16 E1 is associated with cervical cancer progression 17. Furthermore, in 2014, Tsakogiannis *et al*. reported that the A1668G, G2073A, T2169C, T2189C, A2453T, C2454T, A2587T and G2650A mutations of HPV16 *E1* were identified only in high-grade dysplasia cases in a Greek population 14, and this result indicated that those mutations may play an important role in cervical carcinogenesis.

Several studies have reported that the distribution of HPV16 variants is different in different regions and ethnicities 18-21. For example, the A1-A3 (European, EUR) is the predominant variant in the Greek population 18-20, but the A4 (Asian, As) is the predominant variant in the Han Chinese population 21. Thus, in the present study, we aimed to identify HPV16 *E1* mutations in HPV16-positive asymptomatic individuals and HPV16-positive cervical cancer patients and then to evaluate the association of HPV16* E1* mutations with cervical cancer in a Han Chinese population.

## Materials and methods

### Subjects

In the present study, the case group included 161 samples of cervical cancer tissue were gathered from HPV16-positive cervical cancer patients, and 171 samples of cervical exfoliated cells were gathered by fluidbase brush sampling and aseptic cotton sampling from HPV16-positive asymptomatic individuals undergoing routine health check-ups were recruited as the control group. All individuals were recruited at the 3rd Affiliated Hospital of Kunming Medical University during the years 2013 to 2017. The diagnosis of cervical cancer was confirmed by pathological examination according to *CURRENT Diagnosis & Treatment Obstetrics & Gynecology* and FIGO stage (International Federation of Gynaecology and Obstetrics, 2009). Subjects with oncotherapy histories and other malignancies or incomplete clinical data were excluded from the present study. The inclusion criteria for the HPV16-positive asymptomatic individuals were HPV16-positive status, female, and the absence of cervical lesions. All participants were self-reported as ethnically Han and settled in Yunnan for a long time in this study.

### HPV genotyping

For HPV genotyping, the Tellgenplex™ HPV DNA Test which is a suspension bead array method for identifying HPV types was used. The experimental protocol has been described in a previous study 22. Human β-globin was used as an internal control for each reaction.

### Amplification and sequencing of the HPV16* E1* gene

The HPV16 *E1* gene was amplified using a high-fidelity DNA polymerase with the primers 5'-GGACAAGCAGAACCGGA-3' and 5'-CGTCTTGTAATGTCCACTTTTC-3' (2357 bp). The PCR product contained the complete* E1* sequence with a length of 1950 bp. The PCR reactions were performed in a final volume of 25 μl and each PCR mixture contained 1× Q5 PCR buffer (containing Mg2+), 200 μM dNTPs, 0.5 μM sense and antisense primers, 50 ng genomic DNA, and 0.02 U/μl high-fidelity DNA polymerase. The PCR conditions were as follows: an initial denaturation step at 98ºC for 3 min, 35 cycles of denaturation at 98ºC for 10 s, annealing at 64ºC for 30 s, and extension at 72ºC for 90 s, and a final elongation at 72ºC for 2 min. The amplified DNA fragments (including the complete *E1* gene) were sent to Shanghai Sangon Biotech for sequencing after visualization with a 1.0% agarose electrophoresis assay. The primers used for *E1* gene sequencing were 5'-GGACAAGCAGAACCGGA-3', 5'-GCAATTTCACTATCGTCTACTATG-3', 5'-CCAATGCATTTCTTAAATTGTC-3', and 5'-CGTCTTGTAATGTCCACTTTTC-3'.

### Identification and phylogenetic analyses of HPV16 *E1* gene variants

All of the sequences of the HPV16 *E1* sequences were assembled by SeqMan software and aligned by the ClustalW multiple sequence alignment tools included in the Molecular Evolutionary Genetics Analysis (MEGA) v7.0 software package. The HPV16 reference sequence K02718.1 was used to identify mutations in the HPV16 *E1* sequences. Phylogenetic trees of the HPV16* E1* gene were constructed using MEGA 7.0 software by the Maximum likelihood method by bootstrapping with 1000 replications. The reference sequences used to construct the phylogenetic tree were obtained from GenBank, and the accession numbers were HQ644283.1 (A1), HQ644268.1 (A1), HQ644280.1 (A1), HQ644282.1 (A1), AF536179.1 (A2), HQ644236.1 (A3), HQ644248.1 (A4), HQ644251.1 (A4), AF534061.1 (A4), HQ644235.1 (A4), HQ644240.1 (B1), HQ644290.1 (B1), HQ644238.1 (B1), HQ644298.1 (B3), HQ644237.1 (C), HQ644239.1 (C), HQ644249.1 (C), HQ644250.1 (C), AF472509.1 (C), HQ644257.1 (D1), HQ644279.1 (D2), HQ644281.1 (D2), HQ644263.1 (D2), HQ644277.1 (D2), HQ644247.1 (D3), HQ644253.1 (D3), HQ644255.1 (D3), AF402678.1 (D3).

### Statistical analysis

The Student's t-test was used to compare the age distribution between the case and control groups. The frequency of each HPV16 *E1* gene mutation was determined by direct counting. A chi-squared test was performed to analyse the associations among HPV16 variants, HPV16 *E1* mutations and cervical cancer. The statistical analyses were performed using SPSS 13 (Chicago, IL), and *P* values that are less than 0.05 were considered statistically significant.

## Results

### Subject characteristics

In current study, all participants were female, self-reported as ethnically Han and lived in Yunnan for a long time. The case group included 161 HPV16-positive cervical cancer patients and all of them belonged to squamous cell carcinoma (SCC), the age distribution was 46.14 ± 10.14 (mean ± SD). The control group included 171 HPV16-positive asymptomatic individuals, the age distribution was 46.96 ± 9.17 (mean ± SD). There was no significant difference in age between the case and control group (*P*=0.435).

### Distribution of HPV16 variants in the case and control groups

HPV16 variants can be divided into four phylogenetic lineages (A, B, C and D) and 16 sub-lineages: A1-A3 (previously known as EUR variant), A4 (As variant), B1-B4 (AFR1 variant), C1-C4 (AFR2 variant), D1 (NA variant), D2 (AA2 variant), D3 (AA1 variant) and D4. In the current study, the phylogenetic tree constructed by the Maximum likelihood showed the divergence of the HPV16 sub-lineages clearly (Supplementary Figure 1 and 2). There were three HPV16 variants in the case group, namely, A4 (As), A1-A3 (EUR) and D3 (AA1) with distribution frequencies of 68.3%, 29.2% and 2.5%, respectively. However, in the control group, only A4 (As) and A1-A3 (EUR) sub-lineages were found with distribution frequencies of 66.1% and 33.9%. The difference in the distribution of the HPV16 variants between the case group and control group was statistically significant (*P*=0.045).

### HPV16 *E1* gene mutations in the case and control groups

The HPV16 *E1* gene mutations in the case and control groups were shown in Supplementary Table 1; the reference sequence used was K02718.1. In the case group, forty-nine mutations were observed; twenty of these were non-synonymous mutations, and the remaining twenty-nine were synonymous. Moreover, the T921C (T19T), T933A (A23A), T1014G (D50E), A1041G (L59L), C1096G (Q78E), G1163A (G100E), T1200C (A112A), T1366A (C168S), T1407G (S181R), C1426G (Q188E), T1486C (L208L), T1522A (S220T), C1624T (L154L), A1668G (A268A), C1744A (L294M), C2041T (L393L), G2220C (E452D), T2232C (F456F), C2237G (T458S), G2249A (R462K), C2262T (G466G), C2287T (L475L), T2567G (I568S), T2586C (S574S), A2608C (R582R), T2631A (P589P) and G2650A (E596K) mutations were found only in the case group. In the control group, 171 HPV16-positive asymptomatic individuals were accepted into this study. Thirty-one mutations were detected in the control group; nine of these were non-synonymous, and twenty-two were synonymous. Furthermore, the C878A (A5E), T969C (D35D), T1152C (S96S), A1395T (G177G), T1447G (L195V), T1602C (S246S), T2040C (F392F), A2535C (L557L) and A2547G (P561P) mutations were found only in the control group. We found twenty-seven mutations that were statistically significant when the genetic mutation distribution was compared between the case group and control group, and these mutations comprised eleven non-synonymous mutations and sixteen synonymous mutations. Among these mutations, T921C (T19T), A1041G (L59L), C1096G (Q78E), G1163A (G100E), T1200C (A112A), T1366A (C168S), C1426G (Q188E), T1486C (L208L), T1522A (S220T), C1624T (L154L), A1668G (A268A), C1744A (L294M), C2041T (L393L), G2220C (E452D), C2237G (T458S), G2249A (R462K), C2262T (G466G), C2287T (L475L), T2586C (S574S), A2608C (R582R), T2631A (P589P) and G2650A (E596K) were found only in the HPV16 D3 (AA1) sub-lineage; T933A (A23A) and T1014G (D50E) were observed only in the A4 (As) sub-lineage; A2547G (P561P) was found only in the A1-A3 (EUR) sub-lineage; G2160A (R432R) and T2232C(F456F) was found in both the A4 (As) and A1-A3 (EUR) sub-lineages.

### Distribution of HPV16 *E1* gene mutations in the A4 (As), A1-A3 (EUR) and D3 (AA1) sub-lineages

In the HPV16 A4 (As) sub-lineage, fifteen mutations were found in the case group and twelve mutations were found in the control group during the analysis of those sequences. In the case group, seven mutations were non-synonymous and eight mutations were synonymous. In the control group, five mutations were non-synonymous and seven mutations were synonymous. The distributions of the T933A (A23A), T1014G (D50E) and G2160A (R432R) mutations were significantly different between the case and control group for the A4 (As) sub-lineage (Table 1). In the analysis of the A1-A3 (EUR) sub-lineage, a total of fifteen mutations were observed in the case group, among which are five non-synonymous mutations and ten synonymous mutations. In addition, a total of twenty-five mutations were found in the control group, eight of these were non-synonymous and seventeen were synonymous. In the comparison of the distribution of these mutations between the case and control groups, we found that the distributions of the T2232C (F456F), G2337A (M491I) and A2547G (P561P) mutations were significantly different (*P*<0.05) (Table 2). For the D3 (AA1) sub-lineage, which occurred only in the case group, a total of twenty-eight mutations with a frequency of 100% were found; except for T1139G (V92G), C1377T (Y171Y), A1842G (I326M), T2254C (L464L), T2343C (F493F) and C2344T (L494L), these mutations occurred only in the D3 (AA1) sub-lineage. Of these mutations, twelve were non-synonymous (Table 3).

## Discussion

Among all HPV16 proteins, HPV16 E1 is the only protein acting as an enzyme for initiating the replication of the viral genome inside host cells 12. Previous studies found that the E1 protein may also play an important role in mitigating the host's ability to defend against viral infection 23. In the present study, we analysed the distribution of different variants, nucleotide sequence mutations and amino acid substitutions in the HPV16 *E1* gene between HPV16-positive cervical cancer patients and HPV16-positive asymptomatic individuals in a Chinese Han population in order to identify some mutations related to cervical cancer.

Recently, an international study showed that the differential distribution of HPV16 sub-lineages played key roles in the development of cervical cancer 24-29. In 2014, Tsakogiannis *et al*. reported that the HPV16 *E1* ORF alone can be used to correctly classify the HPV genome into the major sub-lineages 14, so we identified the distributions of different variants in samples from the case and control groups by constructing phylogenetic trees with the HPV16 *E1* sequences, and we found that there was different in the distribution of the HPV16 variants between the case group and control group (*P*=0.045) and that the D3 (AA1) sub-lineage was found only in the case group. Many previous studies showed that HPV16 D2 and D3 (AA) sub-lineages appear to be more oncogenic than other sub-lineages, which might contribute to the high incidence of cervical cancer 21, 26, 28-33. In the present study, we observed twenty-seven mutations that had significantly different distributions between the case and control groups. Among these mutations, twenty-two occurred only in the D3 (AA1) sub-lineage. This result also indicated that the D3 (AA1) sub-lineage could be more carcinogenic than the other variants and could play an important role in the development of cervical cancer.

In 1993, Yang *et al*. reported that the bovine papilloma virus (BPV) E1 protein acts as an ATP-dependent DNA helicase and plays a central role in efficient DNA replication in papillomaviruses 34. Given the high degree of homology of El proteins among the papillomaviruses, the HPV E1 protein was deemed to have a function similar to that of BPV E1 and further study also confirmed this hypothesis 12, 35, 36. Furthermore, previous reported that the HPV31 E1 protein can be divided into three functional segments: an N-terminal regulatory region that is essential for optimal replication in vivo, the DNA-binding domain that recognizes specific sites in the origin of replication, and a C-terminal enzymatic domain sufficient for self-assembly into hexamers that display ATPase activity and are capable of unwinding short DNA duplexes 12, 37. Among the mutations with statistically significant differences in distribution between the case and control groups in the current study, the T921C (T19T), T933A (A23A), T1014G (D50E), A1041G (L59L), C1096G (Q78E), G1163A (G100E) and T1200C (A112A) mutations are located in the N-terminal region; the T1366A (C168S), C1426G (Q188E), T1486C (L208L), T1522A (S220T), C1624T (L154L), A1668G (A268A) and C1744A (L294M) mutations are located in the DNA-binding domain; and the C2041T (L393L), G2160A (R432R), G2220C (E452D), T2232C (F456F), C2237G (T458S), G2249A (R462K), C2262T (G466G), C2287T (L475L), A2547G (P561P), T2586C (S574S), A2608C (R582R), T2631A (P589P) and G2650A (E596K) mutations are located in the C-terminal enzymatic domain. These mutations, especially non-synonymous mutations, in different domains may cause functional differences in each functional segment, consequently affecting the roles of E1 in the development of cervical cancer.

In 2014, Tsakogiannis *et al*. reported that the A1668G (A268A), G2073A (K403K), T2169C (D435D), T2189C (I442T), A2453T (N530I), C2454T (N530N), A2587T (R575W) and G2650A (E596K) mutations of HPV16 *E1* were identified only in high-grade dysplasia cases in a Greek population 18. In the current study, we also found that A1668G (A268A) and G2650A (E596K) occurred only in the case group, and this finding suggested these two mutations may play key roles in the carcinogenesis of cervical cancer. Furthermore, our results showed that the T921C (T19T), T933A (A23A), T1014G (D50E), A1041G (L59L), C1096G (Q78E), G1163A (G100E), T1200C (A112A), T1366A (C168S), T1407G (S181R), C1426G (Q188E), T1486C (L208L), T1522A (S220T), C1624T (L154L), C1744A (L294M), C2041T (L393L), G2220C (E452D), T2232C (F456F), C2237G (T458S), G2249A (R462K), C2262T (G466G), C2287T (L475L), T2567G (I568S), T2586C (S574S), A2608C (R582R) and T2631A (P589P) mutations were also found only in the case group, however, these mutations were not found in the study by Tsakogiannis *et al*., but they found the G2073A (K403K), T2169C (D435D), T2189C (I442T), A2453T (N530I), C2454T (N530N) and A2587T (R575W) mutations in the high-grade dysplasia cases, which were not found in our study. Genetic differences between different populations leading to the generation of different mutations in the viral genome during coevolution and the difference in sample sizes may both be partly responsible for the disparity between these studies.

By comparing the mutations in the different variants of HPV16 in this study, we found eight mutations only in the A4 (As) sub-lineage, seventeen mutations only in the A1-A3 (EUR) sub-lineage, and twenty-two mutations only in the D3 (AA1) sub-lineage. Thus, these characteristic *E1* mutations can be specific to each sub-lineage. Moreover, we found consistent mutations in the HPV16 sequences were present in different variants too. Furthermore, we found that T933A (A23A), T1014G (D50E), and G2160A (R432R) showed a significant difference in distribution between the case and control groups for the A4 (As) sub-lineage and that T2232C (F456F), G2337A (M491I), and A2547G(P561P) had a statistically significant difference in distribution between the case group and control group for the A1-A3 (EUR) sub-lineage; thus, these mutations could play different roles in the A4 (As) sub-lineage and A1-A3 (EUR) sub-lineage.

The integration of HPV16 genome into the host chromosome at an early stage of the viral life cycle plays a major role in the pathogenesis of cervical cancer, and the integration of the virus usually disrupts the* E1* and/or *E2* open reading frames (ORFs), which causes transcriptional activation of the viral oncogenes *E6* and* E7*. Therefore, the disruption of the *E1* and *E2* ORFs is associated with the progression of cervical intraepithelial neoplasia to invasive cancer^38^. Furthermore, Filho *et al*. reported that the disruption of HPV16 *E1* or *E2* genes could alter HPV expression by affecting the methylation of 3'LCR in 215 39. However, in 2017, Cricca *et al.* reported *E2* gene could be a more suitable target than *E1* to identify integration into host genome in high-grade cervical lesions 40. Thus, we just focused on the relationship between the mutations of *E1* gene and cervical cancer development not the disruption. However, this could be one of the limitations in the current study.

In the present study, we showed that the D3 (AA1) sub-lineage was highly associated with cervical cancer compared with other variants. Moreover, we found that the T933A (A23A), T1014G (D50E), and G2160A (R432R) mutations in the A4 (As) sub-lineage and the T2232C (F456F), G2337A (M491I), and A2547G (P561P) mutations in the A1-A3 (EUR) sub-lineage played important roles in the development of cervical cancer.

## Supplementary Material

Supplementary figures and tables.

## Figures and Tables

**Table 1:** HPV16* E1* gene mutations and amino acid substitutions in the A4 (As) sub-lineage in the case and control groups.

Genome position ^a^	Case (*n*=110)		Control (*n*=113)		*P* Value*	Amino acid ^a^	Case (*n*=110)		Control (*n*=113)		*P* Value*
	Mutation	Frequency (%)	Mutation	Frequency (%)			Mutation	Frequency (%)	Mutation	Frequency (%)	
T933A	4	3.6	0	0.0	0.041	A23A	0	0.0	0	0.0	-
T1014G	4	3.6	0	0.0	0.041	D50E	4	3.6	0	0.0	0.041
T1139G	110	100.0	113	100.0	-	V92G	110	100.0	113	100.0	-
C1377T	11	10.0	12	10.6	0.879	Y171Y	0	0.0	0	0.0	-
A1395T	0	0.0	2	1.8	0.161	G177G	0	0.0	0	0.0	-
T1407G	3	2.7	0	0.0	0.077	S181R	3	2.7	0	0.0	0.077
T1447G	0	0.0	1	0.9	0.323	L195V	0	0.0	1	0.9	0.323
G1515A	110	100.0	113	100.0	-	V217V	0	0.0	0	0.0	-
T1643C	6	5.5	4	3.5	0.490	L260S	6	5.5	4	3.5	0.490
A1842G	110	100.0	113	100.0	-	I326M	110	100.0	113	100.0	-
G2160A	9	8.2	26	23.0	0.002	R432R	0	0.0	0	0.0	-
T2200C	6	5.5	4	3.5	0.490	L446L	0	0.0	0	0.0	-
T2232C	1	0.9	0	0.0	0.310	F456F	0	0.0	0	0.0	-
T2254C	19	17.3	16	14.2	0.523	L464L	0	0.0	0	0.0	-
G2337A	110	100.0	113	100.0	-	I491M	110	100.0	113	100.0	-
A2439C	11	10.0	12	10.6	0.879	T525T	0	0.0	0	0.0	-
T2567G	3	2.7	0	0.0	0.077	I568S	3	2.7	0	0.0	0.077

^a^ The reference HPV16 *E1* sequence used was K02718.1 ^*^ *P* values less than 0.05 were considered statistically significant.

**Table 2:** HPV16 *E1* gene mutations and amino acid substitutions in the A1-A3 (EUR) sub-lineage in the case and control groups.

Genome position^ a^	Case (*n*=47)		Control (*n*=58)		*P* Value*	Amino acid^ a^	Case (*n*=47)		Control (*n*=58)		*P* Value*
	Mutation	Frequency (%)	Mutation	Frequency (%)			Mutation	Frequency (%)	Mutation	Frequency (%)	
C878A	0	0.0	3	5.2	0.114	A5E	0	0.0	3	5.2	0.114
T969C	0	0.0	2	3.4	0.199	D35D	0	0.0	0	0.0	-
T1139G	47	100.0	58	100.0	-	V92G	47	100.0	58	100.0	-
T1152C	0	0.0	3	5.2	0.114	S96S	0	0.0	0	0.0	-
T1421C	4	8.5	4	6.9	0.757	I186T	4	8.5	4	6.9	0.757
T1447G	0	0.0	1	1.7	0.366	L195V	0	0.0	1	1.7	0.366
G1515A	4	8.5	4	6.9	0.757	V217V	0	0.0	0	0.0	-
T1602C	0	0.0	2	3.4	0.199	S246S	0	0.0	0	0.0	-
A1634C	4	8.5	2	3.4	0.266	Q257P	4	8.5	2	3.4	0.266
A1842G	14	29.8	10	17.2	0.128	I326M	14	29.8	10	17.2	0.128
A1932C	28	59.6	42	72.4	0.165	E356D	28	59.6	42	72.4	0.165
G1941A	4	8.5	2	3.4	0.266	Q359Q	0	0.0	0	0.0	-
T2019C	4	8.5	2	3.4	0.266	T385T	0	0.0	0	0.0	-
T2040C	0	0.0	2	3.4	0.199	F392F	0	0.0	0	0.0	-
A2158C	6	12.8	5	8.6	0.490	R432R	0	0.0	0	0.0	-
G2160A	0	0.0	1	1.7	0.366	R432R	0	0.0	0	0.0	-
T2232C	4	8.5	0	0.0	0.023	F456F	0	0.0	0	0.0	-
T2233C	6	12.8	5	8.6	0.490	L457L	0	0.0	0	0.0	-
T2301C	16	34.0	19	32.8	0.890	A479A	0	0.0	0	0.0	-
G2337A	0	0.0	6	10.3	0.023	M491I	0	0.0	6	10.3	0.023
T2343C	0	0.0	2	3.4	0.199	F493F	0	0.0	0	0.0	-
C2344T	5	10.6	9	15.5	0.465	L494L	0	0.0	0	0.0	-
T2376G	17	36.2	19	32.8	0.714	S504S	0	0.0	0	0.0	-
A2535C	0	0.0	2	3.4	0.199	L557L	0	0.0	0	0.0	-
A2547G	0	0.0	7	12.1	0.014	P561P	0	0.0	0	0.0	-
T2595G	17	36.2	19	32.8	0.714	P577P	0	0.0	0	0.0	-

^a^ The reference HPV16 *E1* sequence used was K02718.1 ^*^ *P* values less than 0.05 were considered statistically significant.

**Table 3:** HPV16 *E1* gene mutations and amino acid substitutions in the D3 (AA1) sub-lineage in the case and control groups.

Genome position^ a^	Case (*n*=4)		Amino acid^ a^	Case (*n*=4)	
	Mutation	Frequency (%)		Mutation	Frequency (%)
T921C	4	100.0	T19T	0	0.0
A1041G	4	100.0	L59L	0	0.0
C1096G	4	100.0	Q78E	4	100.0
T1139G	4	100.0	V92G	4	100.0
G1163A	4	100.0	G100E	4	100.0
T1200C	4	100.0	A112A	0	0.0
T1366A	4	100.0	C168S	4	100.0
C1377T	4	100.0	Y171Y	0	0.0
C1426G	4	100.0	Q188E	4	100.0
T1486C	4	100.0	L208L	0	0.0
T1522A	4	100.0	S220T	4	100.0
C1624T	4	100.0	L154L	0	0.0
A1668G	4	100.0	A268A	0	0.0
C1744A	4	100.0	L294M	4	100.0
A1842G	4	100.0	I326M	4	100.0
C2041T	4	100.0	L393L	0	0.0
G2220C	4	100.0	E452D	4	100.0
C2237G	4	100.0	T458S	4	100.0
G2249A	4	100.0	R462K	4	100.0
T2254C	4	100.0	L464L	0	0.0
C2262T	4	100.0	G466G	0	0.0
C2287T	4	100.0	L475L	0	0.0
T2343C	4	100.0	F493F	0	0.0
C2344T	4	100.0	L494L	0	0.0
T2586C	4	100.0	S574S	0	0.0
A2608C	4	100.0	R582R	0	0.0
T2631A	4	100.0	P589P	0	0.0
G2650A	4	100.0	E596K	4	100.0

^a^ The reference HPV16 *E1* sequence used was K02718.

## Abbreviations

HPV16: human papillomavirus type 16
LCR: long control region
EUR: European
As: Asian
NA: North American
AA1: Asian-American 1
AA2: Asian-American 2
AFR1: African-1
AFR2: African-2
ORF: open reading frame
