# Supplementary material for: Human Papillomavirus Type 16 E1 Mutations Associated with Cervical Cancer in a Han Chinese Population
Source: Int J Med Sci. 2019 Jun 24;16(7):1042–9. doi: 10.7150/ijms.34279 (PMC6643129; doi:10.7150/ijms.34279)
Supplement: Supplementary file 1 — Supplementary figures and tables. [file ijmsv16p1042s1.pdf]

Supplementary Table 1. HPV16 *E1* gene mutations and amino acid substitutions in the case and control groups.

Genome position [a]	Case (*n*=161)		Control (*n*=171)		*P* Value*	Amino acid [a]	Case (*n*=161)		Control (*n*=171)		*P* Value*
	Mutation	Frequency (%)	Mutation	Frequency (%)			Mutation	Frequency (%)	Mutation	Frequency (%)	
C878A	0	0.0	3	1.8	0.091	A5E	0	0.0	3	1.8	0.091
T921C	4	2.5	0	0.0	0.038	T19T	0	0.0	0	0.0	-
T933A	4	2.5	0	0.0	0.038	A23A	0	0.0	0	0.0	-
T969C	0	0.0	2	1.2	0.169	D35D	0	0.0	0	0.0	-
T1014G	4	2.5	0	0.0	0.038	D50E	4	2.5	0	0.0	0.038
A1041G	4	2.5	0	0.0	0.038	L59L	0	0.0	0	0.0	-
C1096G	4	2.5	0	0.0	0.038	Q78E	4	2.5	0	0.0	0.038
T1139G	161	100.0	171	100.0	-	V92G	161	100.0	171	100.0	-
T1152C	0	0.0	3	1.8	0.091	S96S	0	0.0	0	0.0	-
G1163A	4	2.5	0	0.0	0.038	G100E	4	2.5	0	0.0	0.038
T1200C	4	2.5	0	0.0	0.038	A112A	0	0.0	0	0.0	-
T1366A	4	2.5	0	0.0	0.038	C168S	4	2.5	0	0.0	0.038
C1377T	15	9.3	12	7.0	0.444	Y171Y	0	0.0	0	0.0	-
A1395T	0	0.0	2	1.2	0.169	G177G	0	0.0	0	0.0	-
T1407G	3	1.9	0	0.0	0.073	S181R	3	1.9	0	0.0	0.073
T1421C	4	2.5	4	2.3	0.931	I186T	4	2.5	4	2.3	0.931
C1426G	4	2.5	0	0.0	0.038	Q188E	4	2.5	0	0.0	0.038
T1447G	0	0.0	2	1.2	0.169	L195V	0	0.0	2	1.2	0.169
T1486C	4	2.5	0	0.0	0.038	L208L	0	0.0	0	0.0	-
G1515A	114	70.8	117	68.4	0.637	V217V	0	0.0	0	0.0	-

T1522A	4	2.5	0	0.0	0.038	S220T	4	2.5	0	0.0	0.038
T1602C	0	0.0	2	1.2	0.169	S246S	0	0.0	0	0.0	-
C1624T	4	2.5	0	0.0	0.038	L154L	0	0.0	0	0.0	-
A1634C	4	2.5	2	1.2	0.369	Q257P	4	2.5	2	1.2	0.369
T1643C	6	3.7	4	2.3	0.460	L260S	6	3.7	4	2.3	0.460
A1668G	4	2.5	0	0.0	0.038	A268A	0	0.0	0	0.0	-
C1744A	4	2.5	0	0.0	0.038	L294M	4	2.5	0	0.0	0.038
A1842G	128	79.5	123	71.9	0.108	I326M	128	79.5	123	71.9	0.108
A1932C	28	17.4	42	24.6	0.109	E356D	28	17.4	42	24.6	0.109
G1941A	4	2.5	2	1.2	0.369	Q359Q	0	0.0	0	0.0	-
T2019C	4	2.5	2	1.2	0.369	T385T	0	0.0	0	0.0	-
T2040C	0	0.0	2	1.2	0.169	F392F	0	0.0	0	0.0	-
C2041T	4	2.5	0	0.0	0.038	L393L	0	0.0	0	0.0	-
A2158C	6	3.7	5	2.9	0.683	R432R	0	0.0	0	0.0	-
G2160A	9	5.6	27	15.8	0.003	R432R	0	0.0	0	0.0	-
T2200C	6	3.7	4	2.3	0.460	L446L	0	0.0	0	0.0	-
G2220C	4	2.5	0	0.0	0.038	E452D	4	2.5	0	0.0	0.038
T2232C	5	3.1	0	0.0	0.020	F456F	0	0.0	0	0.0	-
T2233C	6	3.7	5	2.9	0.683	L457L	0	0.0	0	0.0	-
C2237G	4	2.5	0	0.0	0.038	T458S	4	2.5	0	0.0	0.038
G2249A	4	2.5	0	0.0	0.038	R462K	4	2.5	0	0.0	0.038
T2254C	23	14.3	16	9.4	0.163	L464L	0	0.0	0	0.0	-
C2262T	4	2.5	0	0.0	0.038	G466G	0	0.0	0	0.0	-
C2287T	4	2.5	0	0.0	0.038	L475L	0	0.0	0	0.0	-

T2301C	16	9.9	19	11.1	0.728	A479A	0	0.0	0	0.0	-
G2337A	110	68.3	119	69.6	0.803	M491I	110	68.3	119	69.6	0.803
T2343C	4	2.5	2	1.2	0.369	F493F	0	0.0	0	0.0	-
C2344T	9	5.6	9	5.3	0.895	L494L	0	0.0	0	0.0	-
T2376G	17	10.6	19	11.1	0.872	S504S	0	0.0	0	0.0	-
A2439C	11	6.8	12	7.0	0.947	T525T	0	0.0	0	0.0	-
A2535C	0	0.0	2	1.2	0.169	L557L	0	0.0	0	0.0	-
A2547G	0	0.0	7	4.1	0.009	P561P	0	0.0	0	0.0	-
T2567G	3	1.9	0	0.0	0.073	I568S	3	1.9	0	0.0	0.073
T2586C	4	2.5	0	0.0	0.038	S574S	0	0.0	0	0.0	-
T2595G	17	10.6	19	11.1	0.872	P577P	0	0.0	0	0.0	-
A2608C	4	2.5	0	0.0	0.038	R582R	0	0.0	0	0.0	-
T2631A	4	2.5	0	0.0	0.038	P589P	0	0.0	0	0.0	-
G2650A	4	2.5	0	0.0	0.038	E596K	4	2.5	0	0.0	0.038

[a] The reference HPV16 *E1* sequence used was K02718.1

[*] *P* values less than 0.05 were considered statistically significant.

Supplementary Figure 1

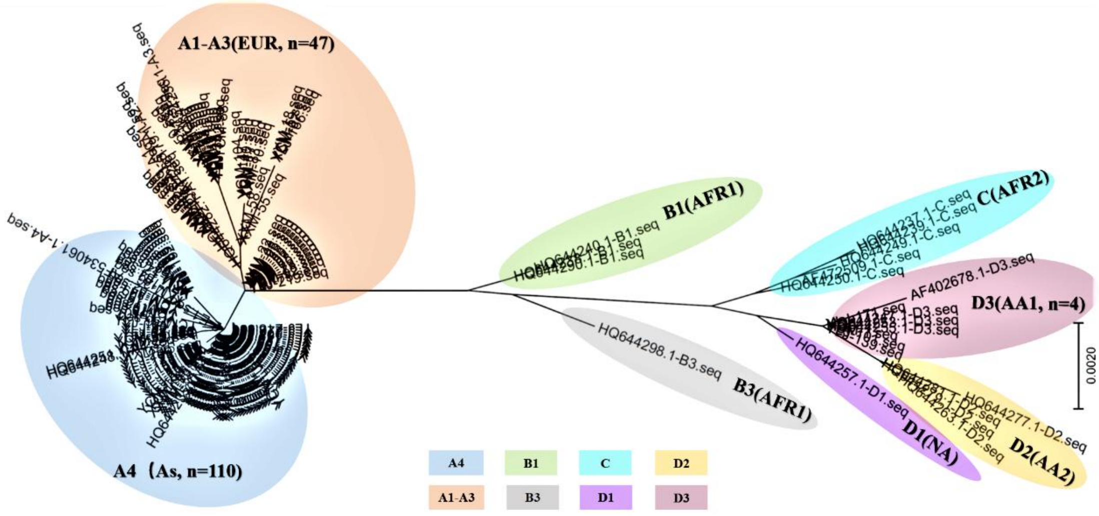

Supplementary Figure 1. Phylogenetic tree based on the HPV16 *E1* gene sequences from HPV16-positive cervical cancer patients, corresponding to the K02718.1 reference sequence.

Supplementary Figure 2

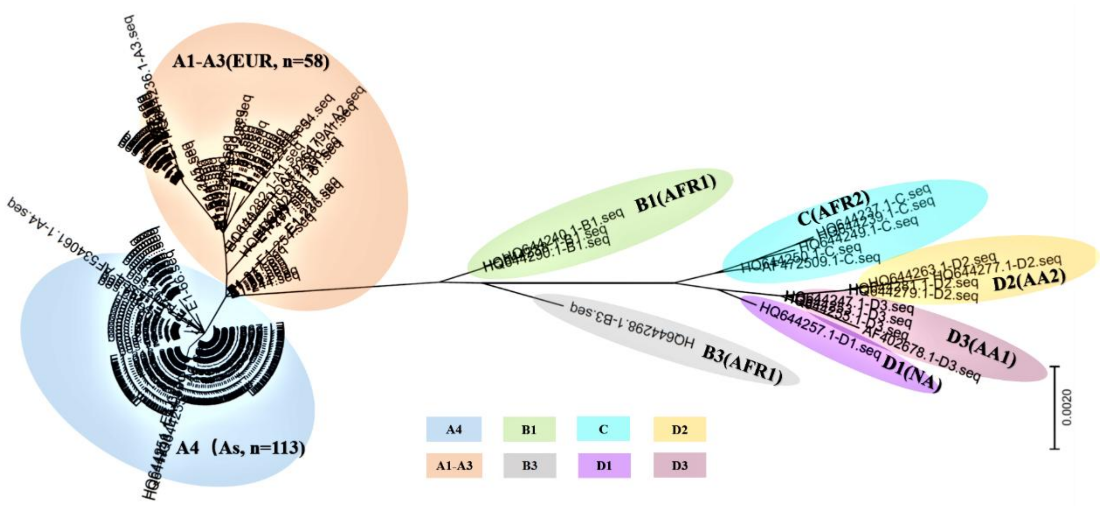

Supplementary Figure 2. Phylogenetic tree based on the HPV16 *E1* gene sequences from HPV16-positive asymptomatic individuals, corresponding to the K02718.1 reference sequence.
